# HIV seroprevalence and its effect on outcome of moderate to severe burn injuries: A Ugandan experience

**DOI:** 10.1186/1752-2897-5-8

**Published:** 2011-06-09

**Authors:** Phillipo L Chalya, Robert Ssentongo, Ignatius Kakande

**Affiliations:** 1 Department of Surgery, Weill- Bugando University College of Health Sciences, Mwanza, Tanzania; 2 Reconstructive and Plastic Surgery, Mulago Hospital Complex, Kampala, Uganda; 3 Department of Surgery, Makerere University, Kampala, Uganda

**Keywords:** HIV seroprevalence, outcome, burn injuries, Uganda

## Abstract

**Background:**

HIV infection in a patient with burn injuries complicates the care of both the patient and the treating burn team. This study was conducted to establish the prevalence of HIV among burn patients in our setting and to compare the outcome of these patients who are HIV positive with those who are HIV negative.

**Methods:**

This was a prospective cohort study involving burn injury patients admitted to Mulago Hospital between November 2005 and February 2006. Patients were stratified into HIV positive (exposed) group and HIV-negative (unexposed) group. Data was collected using a pre-tested coded questionnaire and analyzed using SPSS statistical computer software version 11.5.

**Results:**

Of the 130 patients included in the study, 17 (13.1%) patients tested HIV positive and this formed the study (exposed) group. The remaining 113 patients (86.9%) formed the control (unexposed) group. In the HIV positive group, females outnumbered males by a ratio of 1.4:1 and the mean age was 28.4 ± 21.5 years (range 3 months-34 years). 64.7% of HIV positive patients reported to have risk factors for HIV infection. Of these, multiple sexual partners [Odds Ratio 8.44, 95% C.I. (3.87-143.23), P = 0.011] and alcoholism [Odds Ratio 8.34, 95% C.I. (5.76-17.82), P = 0.002] were found to be independently and significantly associated with increased risk to HIV infection. The mean CD4 count for HIV positive and HIV negative patients were 394 ± 328 cells/μL and 912 ± 234 cells/μL respectively which is statistically significant (P = 0.001). There was no difference in the bacteria cultured from the wounds of HIV positive and negative patients (P = 0.322). Patients with clinical signs of sepsis had lower CD4+ counts compared to patients without sepsis (P < 0.001). ). Skin grafting was carried out in 35.3% of HIV negative patients and 29.4% of HIV positive patients with no significant difference in skin graft take and the degree of healed burn on discharge was the same (P = 0.324). There was no significant difference in hospital stay between HIV positive and negative patients (P = 0.674). The overall mortality rate was 11.5%. Using multivariate logistic regression analysis, mortality rate was found to be independently and significantly related to the age of the patient, HIV positive with stigmata of AIDS, CD4 count, inhalation injury, %TBSA and severity of burn (p-value < 0.001).

**Conclusion:**

HIV infection is prevalent among burn injury patients in our setting and thus presents an occupational hazard to health care workers who care for these patients. All burn health care workers in this region need to practice universal precautions in order to reduce the risk of exposure to HIV infection and post-exposure prophylaxis should be emphasized. The outcome of burn injury in HIV infected patients is dependent upon multiple variables such as age of the patient, inhalation injury and %TBSA and not the HIV status alone.

## Background

Burn injuries continue to be the most common public health problem worldwide and it is associated with high morbidity and mortality both in developed and developing countries [1]. In Uganda, burn injuries continue to be one of the leading causes of morbidity and mortality and at Mulago Hospital, burn injuries are the single commonest indication for admission reported in the surgical wards and burn unit [2-4].

The management of burn patients poses a challenge to health care workers in developing countries where resources for caring these patients are limited [5]. HIV infection in a patient with burn injury complicates the care of both the patient and the treating burn team. In the patient, HIV slows wound healing and increases the complications seen with burns. For the treating team, HIV is a hazard that infects the large volume of bodily fluids to which the burn team is potentially exposed [5,6]. The risk of exposure to HIV in the management of burn injuries depends on the underlying prevalence of infection in the general population. In developing nations including Africa, the population prevalence of HIV in some groups can be as high as 30% [7]. In trauma population including burn injuries, the prevalence of HIV seropositivity have been reported to run as high as 19% and thus presents an occupational hazard to health care workers who care for these patients [8].

Universal precautions have been reported to reduce the rate of HIV transmission; however, studies have indicated poor compliance to universal precautions in developing countries where resources for caring of these patients are limited [5,8-12]. Thus, the risk of exposure to HIV infection among health care workers who care for burn patients in our setting is high.

The current seroprevalence of human immunodeficiency virus (HIV) in burn population in our setting is unknown. Establishing the seroprevalence of HIV in burn patients is vital for education and post-exposure prophylaxis [13].

Data from previous studies in Mulago Hospital have reported that the outcome of burn injury patients has not changed much for the last three decades despite improvement in burn care and establishment of burn unit [2-4]. The reason for this state of affairs is not known. The impact of HIV infection on the outcome of burn patients has not been studied at Mulago Hospital and Uganda in general. This study intended to establish the prevalence of HIV among burn injury patients in our setting and to compare the outcome of these patients who are HIV positive with those who are HIV negative.

## Methods

This was a prospective cohort study which was conducted at the Accident and Emergency department of Mulago Hospital, Kampala, Uganda over a four month period from November 2005 to February 2006. Mulago Hospital is located in the capital city Kampala. This is the largest hospital in the country and has a bed capacity of 1500. The hospital serves as a national referral hospital and a teaching hospital for Makerere University and other paramedical staff. Burn unit of Mulago hospital was established in 2004 and has a bed capacity of twelve beds

The study subjects included all burn injury patients of all age groups and gender and who consented for the study and HIV testing. Patients were stratified into HIV positive (exposed) and HIV negative (unexposed). Patients who failed to provide information and had no relative nearby and those who failed to consent for HIV testing were excluded from the study. Patients who were still in the ward at the end of study period were also excluded from the study. Patients admitted 7 days post-burn and patients admitted for post-burn reconstructive surgery were also excluded. Recruitment of patients was done at the emergency department and in the surgical wards after primary and secondary surveys done by the admitting surgical team.

The study was carried out after the approval of the department of Surgery, Faculty of Medicine Research and Ethics Committee and Uganda National Council of Science and Technology. After informed consent to participate in the study and to be tested for HIV, all patients who met the inclusion criteria were consecutively enrolled into the study. All patients were precounselled by a professional HIV counselor according to current guidelines in the hospital. The patients were offered to be informed of the result of the HIV test. When personal consent was not possible due to the nature of the injuries or age below 18 years, consent was sought from the family. Patients were assured that the result would not affect their management and were given the choice to know their results. To prevent bias the medical and nursing staff were not informed about the results of the HIV test. All patients were treated according to Mulago Hospital protocol which, given the very high prevalence of HIV in general population, assumes that all patients are potentially infected with HIV and staff anticipate HIV related complications and problems in nursing care as a part of daily routine.

On admission, a sample of blood was taken from the patients and used to carry out HIV serology test, CD4+ cell count and complete blood count. A rapid antibody test (Uganda HIV Rapid Test Algorithm) was used to screen for HIV infection to all patients aged above 18 months. If two or more blood test were reactive on rapid antibody test, then the results were conformed using ELISA test. Polymerase Chain Reaction (PCR) was used to screen children under the age of 18 months. In addition, wound swabs were also taken for culture and sensitivity on admission and then on day ten after admission.

Patients were stratified into HIV positive (exposed) and HIV negative (non-exposed). The study group (exposed) comprised of those who tested positive for HIV and the control group (non-exposed) was made up of unmatched patients known to be negative. The main variables known to influence outcome from burn injury were matched. These were sex, age, type of burn, %TBSA and inhalation injury

All recruited patients were managed according to advanced trauma life support [ATLS]. The total body surface area (TBSA) was assessed using a "Lund-Browder Chart". Total body surface area (TBSA) is an assessment measure of burns of the skin, burned surface area is calculated as a percentage of total body surface area to determine burn size. The burn depth was assessed clinically by observation of the burn wound. The diagnosis of burn wound infection was based on careful clinical examination (purulent discharge from the wound + signs of inflammation) and identification of micro-organisms from the area of the burn wound suspected of being infected. Stigmata of AIDS were defined as occurrence of opportunistic infection and tumors in patients infected with HIV.

Patients with severe burns were subsequently admitted in the burn unit and those with moderate burns were admitted to the parent admitting surgical wards. Associated injuries were managed appropriately according to the type of injury. Patients were followed up until discharge or death. HIV positive patients were referred to the Care and Treatment Clinic (CTC) after post- counseling. Data was collected using a pre-tested coded questionnaire. Data administered in the questionnaire included; socio-demographic data e.g. age, sex, occupation; circumstance of injury, characteristics of injury and HIV status and outcome measures. Outcome was measured in terms of survival, treatment parameters, complications and the length of hospital stay.

### Statistical analysis

Data collected were entered into a computer and analyzed using SPSS software version 11.5. Data were summarized in form of proportions and frequent tables for categorical variables. Means, median, mode, standard deviation were used to summarize continuous variables. Chi-square test was used to test for significance of associations between the predictor and outcome variables in the categorical variables. Odds ratio was calculated to test for strength of association between predictor variables. Student t-test was used to test for significance of associations between the predictor and outcome variables in the continuous variables. Significance was defined as a p-value of less than 0.05. Multivariate logistic regression analysis was used to determine predictor variables that are associated with outcome

## Results

### Patient characteristics

During the period under study, a total of 152 burn injury patients were admitted to the A & E surgical ward. Of these, 22 patients were excluded from the study due to failure to meet the inclusion criteria. Thus, a total of 130 burn injury patients were studied. Generally, 70 (53.8%) were males and 60 (46.2%) were females (Male to Female ratio = 1.2:1). The patients' ages ranged from 3 months to 80 years (14.00 ± 15.98). Children up to 15 years were 86(66.2%); of whom 60(64.8%) were under the age of 5 years. Only 4 patients (9.1%) were aged 65 years and above.

Of the 130 patients included in the study, 17 (13.1%) patients tested HIV positive and this formed the study (exposed) group. The remaining 113 patients (86.9%) formed the control (unexposed) group. In the HIV positive group, 7 (41.2%) were males and 10 (58.8%) were females (Male: Female ratio = 1:1.4). The ages of HIV positive patients ranged from 3 months to 34 years (28.4 ± 21.5). Of these, twelve patients (70.6%) were aged above 15 years. The remaining 5 patients (29.4%) were children aged 15 years and below. Of the 17 patients with HIV infection, eleven (64.7%) patients reported to have risk factors for HIV infection. Of these, multiple sexual partners [Odds Ratio 8.44, 95% C.I. (3.87-143.23), P = 0.011] and alcoholism [Odds Ratio 8.34, 95% C.I. (5.76-17.82), P = 0.002] were found to be independently and significantly associated with increased risk to HIV infection. Six patients (35.3%) had stigmata of AIDS. Majority of HIV positive patients (58.8%) had clinical stage I. The TBSA for HIV positive patients ranged from 5% to 50% with the median and mean of 16.75 and 18.72%±9.81 respectively and majority of them (94.1%) had %TBSA below 40%. (Table 1)

**Table 1:** Patient's characteristics according to HIV status

Patient characteristics	HIV positive	HIV negative	P-value
Age group			
< 15 years	5 (29.4%)	81(71.7%)	
≥15 years	12(70.6%)	32(28.3%)	0.012
Gender			
Male	7 (41.2%)	63(55.8%)	
Female	10(58.8%)	50(44.2%)	0.794
Type of burn			
Scald	7(41.2%)	52(46.0%)	
Flame	7(41.2%)	47(41.6%)	
Chemical	2(11.8%)	8(7.1%)	
Electrical	1(5.9%)	5(4.4%)	
Contact	-	1(0.9%)	0.065
Inhalation injury			
Present	2 (11.8%)	4 (3.5%)	
Not present	15 (88.2%)	109 (96.5%)	0.001
%TBSA			
< 40%	16(94.1%)	106(93.8%)	
≥40%	1(5.9%)	7(6.2%)	0.002
Risk factors for HIV infection			
Present	11 (64.7%)	23 (20.4%)	
Absent	6 (35.3%)	90 (79.6%)	0.897
Stigmata of HIV/AIDS			
Present	6(35.3%)	0 (0%)	
Absent	11(64.7%)	113(100%)	0.017
Clinical staging of AIDS			
Stage I	10(58.8%)	-	
Stage II	1(5.9%)	-	
Stage III	1(5.9%)	-	
Stage IV	4(23.5%)	-	
CD4 count			
Mean ± S.D cells/μL	394 ± 328	912 ± 234	0.001
< 200 cells/μL	5 (29.4%)	1(0.9%)	
≥200 cells/μL	12 (70.6%)	112 (99.1%)	0.000
Haematological results (mean ± S.D)			
Hemoglobin	12.6 ± 4.2	12.4 ± 4.4	0.076
Packed cell volume (PCV)	39.6 ± 8.4	39.8 ± 7.8	0.764
Platelets	294 ± 112	354 ± 168	0.351
Total white blood cells	9.4 ± 4.8	14.0 ± 5.1	0.054

### Haematological results

Hemoglobin, platelets and PCV levels showed no statistically significant differences in relation to HIV status (P > 0.001). The mean CD4 count for HIV positive and HIV negative patients were 394 ± 328 cells/μL and 912 ± 234 cells/μL respectively. This difference was statistically significant (P = 0.001). Five patients (29.4%) in the group of HIV infected patients had CD4 count ≤ 200 cells/μL and the remaining 12 (70.6%) HIV positive patients had CD4 count > 200 cells//μL.

Both HIV status and TBSA were found to be independent predictors of CD4 count (P = 0.021 and P = 0.0011, respectively).

### Microbiological results

The rate of burn wound infection for HIV positive patients on admission and on 10^th ^day were 27.6% and 35.8% respectively and the rate of infection for HIV negative patients on admission and on 10th day were 22.4% and 28.2% respectively. No significant difference was found between infection rate and HIV status (P = 0.218). There was no difference in the bacteria cultured from the wounds of HIV positive and negative patients (P = 0.322). ). Patients with clinical signs of sepsis had lower CD4+ counts compared to patients without sepsis (P < 0.001). *Staphylococcus aureus *were more common on admission wound swabs, with *Pseudomonas aeruginosa *becoming more evident after 10th day. MRSA was detected in 21.3% of *Staphylococcus aureus*.

### Treatment parameters

The number of patients receiving IV fluids, blood transfusion and antibiotics did not differ significantly in the two groups (P = 0.342). Skin grafting was carried out in 35.3% of HIV negative patients and 29.4% of HIV positive patients with no significant difference in skin graft take and the degree of healed burn on discharge was the same (P = 0.324). (Table 2).

**Table 2:** Outcome of treatment according to HIV status

Outcome (dependent ) variables	HIV positive (N = 17)	HIV negative (N = 113)	P-value
Infection rate			
• On admission	27. 6%	22.4%	
• On 10^th ^day	35.8%	28.2%	0.218
% Healing at discharge (mean)	84%	92%	0.059
Skin grafting done	5(29.4%)	6(5.3%)	0,564
Skin graft take %	90%	91%	0.324
Mean length of hospital stay (days)	29.5 ± 21.8	23.84 ± 22.42.	0.674
Mortality	29.4%	8.8%	0.014

### Outcome according to HIV status

The mean length of hospital stay for HIV positive patients with stigmata of AIDS and those without stigmata of AIDS were 22.18 ± 14.04 and 21.76 ± 18.15 respectively, and those with HIV negative patients was 23.84 ± 22.42. There was no significant difference in hospital stay between HIV positive and negative patients (P = 0.674). Fifteen patients died giving an overall mortality of 11.5%. Five out of 17 (29.4%) HIV positive patients died compared to 10 out of 113 (8.8%) HIV negative patients which is significant (P = 0.014). The mortality rate for HIV positive patients with stigmata of AIDS was 83.3% compared to 8.1% for HIV positive patients without stigmata of AIDS. These differences were statistically significant (P = 0.032). Mortality rate for HIV infected patients with CD4 count < 200 cells/μL was 80% (4 out of 5 patients died in this group) which is significantly higher compared to 8.3% (1 out of 12 patients) for HIV positive patients with CD4 count ≥ 200 cells/μL (P = 0.012). There was no significant difference in terms of outcome between HIV positive patients with CD 4 count ≥ 200 cells/ μl and HIV negative patients (P = 0.643). Four of 17 (23.5%) patients of the HIV positive patients died of sepsis compared to 6 of 113 (5.3%) of the HIV negative patients (P = 0.003).

The predictors of death according to univariate and multivariate logistic regression analysis are shown in table 3.

**Table 3:** Predictors of death according to Univariate and Multivariate analysis

	Univariate analysis			Multivariate analysis		
	
Independent variables	Odds ratio	**95% C.I**.	P-value	Odds ratio	**95% C.I**.	P-value
Age	1.35	1.04-3.09	0.012	8.44	1.62-43.87	0.011
Sex	5.21	3.87-9.11	0.234	2.88	2.01-13.87	0.765
Type of burn	2.09	1.34-6.76	0.012	4.76	3.078-3.98	0.432
%TBSA	4.86	3.11-12.23	0.003	87.00	3.82-96.88	0.000
Inhalation injury	2.43	1.32-56.90	0.001	15.98	2.27-17.30	0.005
HIV status	1.23	1.07-3.42	0.010	1.61	1.11-1.98	0.077
Stigmata of HIV/AIDS	2.98	2.12-6.76	0.000	25.09	2.07-30.11	0.001
Clinical staging of AIDS	1.54	0.89- 2.12	0.034	3.23	1.43-4.09	0.056
CD4 count	2.12	1.54-34.56	0.012	1.34	1.10-1.43	0.001

## Discussion

The prevalence of HIV seropositivity in some trauma population including burns has been reported to be higher than in general population and thus presents an occupational hazard to healthcare workers who care for these patients [8]. The overall seroprevalence of HIV infection in our study was 13.1% that is higher than that in the general population in Uganda (6.5%) [14]; these may be attributed to high percentage of the risk factors for HIV infection reported in the present study population. Similar observations were also reported by other studies [15-17]. This implies that health care workers who care for burn patients are at high risk of HIV transmission due to frequent contact with body fluids starting from the Acute & Emergency department to wards and in operating theatres. The overall HIV seroprevalence in our study may actually be an underestimate and the magnitude of the problem may not be apparent because many cases (22 patients) were excluded from the study due to failure to meet the inclusion criteria.

Our seroprevalence of HIV in the present study was found to be slight higher than that reported in Malawi reflecting difference in the overall prevalence of HIV infection in general population from one country to another [6]. The prevalence of HIV infection in adult above 15 years and in children 15 years and below in the present study was comparable to that reported in Malawian study [6]. The HIV prevalence in children ≤15 years of age is most likely caused by vertical transmission or by blood transfusion for malaria-related anemia

Our study showed a female preponderance which is in contrast to other studies which reported male preponderance [6,18]. We could not establish the reasons for this gender difference.

Large number of HIV positive patients with clinical stage I (asymptomatic HIV infected patients) in our study was also reported by other studies [6,19]. Large number of asymptomatic HIV infected burn injury patients in our study highlights the importance of prevention and strict use of universal precautions among health care workers who care for these patients.

The Centers for Disease Control and Prevention (CDC) recommends routine screening for HIV with informed consent, for patients between 15 to 54 years of age in regions of high HIV prevalence [20]. In Mulago hospital, burn injury patients are not routinely screened and therefore little is known about the magnitude of the problem and the risk of HIV transmission among health care workers who care for these patients.

Compliance with universal precautions has been reported to reduce the risk of HIV transmission among trauma (including burn) health care workers. However, recent studies have shown that compliance with universal barrier precautions in the high-risk setting of emergency rooms, surgical suites, and critical care units in developing countries like Uganda is less than optimal [9-12]. This observation may be attributed to lack of knowledge of universal precautions and limited resources.

Compliance with universal precautions thus becomes an important issue, and determining the reasons for failure to comply with universal precautions becomes an important priority as well [9,11,12].

Human immunodeficiency virus infection has been reported to have an impact on the outcome of burn injuries [5,6,19]. In the present study, HIV positive individuals had significantly lower CD4 counts than HIV negative patients; the later been attributed to the effect of burn alone and HIV-infected burn patients be immunosuppressed both from the HIV infection as well as from the burn itself, both factors adding increased risk of death in particular of sepsis. In our study, both HIV status and TBSA were found to be independent predictor of CD4 count which is in agreement with James *et al *[6] in Malawi. HIV positive patients with burns are more likely to be more profoundly immunosuppressed compared with HIV negative burn patients, caused by the HIV infection as well as by (the extent of) the burn. The TBSA of the burn remains the most important predictor of outcome regardless of HIV status [6].

Infection rates and bacterial profile between HIV positive patients and HIV negative patients in the present study revealed no significant difference. Similar trend of infection rates and bacterial profile was also reported by other studies [6,21]. Despite the above observations, the authors of the present study still believe that burn wound sepsis still contributes significantly to high morbidity and mortality among burn injury patients.

Impaired survival of skin grafts has been noted in human immunodeficiency virus (HIV) infected patients, but the reason is not known. Alterations in inflammatory response, which might be recorded as an imbalance in cytokine production, have been implicated [18,22,23]. The present study showed no significant difference in skin graft take and the degree of healed burn on discharge. This is in agreement with the report from other studies [6,22] but in contrast to the report of Delaney *et al *[21] who described delayed wound healing, opportunistic infections and skin graft loss in HIV-infected burns patients.

The present study revealed no significant difference in the time spent as an inpatient between those who were HIV positive and the controls. Similar findings were also reported by other studies [6,19].

Due to the poor socio-economic conditions in Uganda, the duration of inpatient stay for our patients may be longer than expected. However this is a problem in both the study and control group.

Our mortality rate for HIV positive was significantly higher than that of HIV negative patients (29.4% versus 8.8%). These mortality figures were comparable to that reported by one study [6]. The mortality rate for HIV positive patients with stigmata of AIDS in this study was 83.3% lower that reported in a study in South Africa (100%) [19]. There was no significant difference between HIV positive patients with CD 4 count ≥ 200 cells/ μl and HIV negative patients. This observation reflects that the prognosis of HIV positive patients with CD 4 count ≥ 200 cells/ μl is similar to that of HIV negative patients. Our CD4 profile and its influence on the outcome of burned patients were comparable to that reported in the Malawian study [6].

Despite limited follow-up time and difficult to diagnose HIV infection in its early stage ("window period"), the study has shown that the outcome in burn patients is dependent upon multiple variables and not the underlying immunodeficiency (i.e. HIV seropositivity and CD4+ cell count) alone. Other factors influencing the outcome of trauma patients include age of the patient, inhalation injury, TBSA etc.

## Conclusion

This study has shown that HIV infection is prevalent among burn injury patients in our setting and thus presents an occupational hazard to health care workers who care for these patients. Therefore, all burn health care workers in this region need to practice universal barrier precautions in order to reduce the risk of exposure to HIV infection. Post-exposure prophylaxis should be emphasized. HIV positive patients with CD4+ count ≥200cells/μl have similar prognosis as HIV negative patients and therefore should be treated the same way. The study has also shown that the outcome of burn injury in HIV infected patients is dependent upon multiple variables and not the underlying immunodeficiency alone. The age of the patient, inhalation injury and %TBSA remain the most common factors affecting the outcome of burn injury patients regardless of HIV status.

## Competing interests

The authors declare that they have no competing interests.

## Authors' contributions

PLC conceived the study and did the literature search, coordinated the write-up, editing and submission of the article. RS participated in the literature search, writing of the manuscript, editing and supervised the study. IK coordinated the write-up, editing and supervised the study. All the authors read and approved the final manuscript

## Authors' information

PLC: Consultant general surgeon and Lecturer, Department of Surgery, Well Bugando University College of Health Sciences. RS: Consultant Reconstructive and Plastic Surgeon, Mulago Hospital Complex, Kampala, Uganda and IK: Professor, Department of Surgery, Makerere University, Kampala, Uganda.
